# Rhenium Corrole Dimers: Electrochemical Insights into the Nature of the Metal–Metal Quadruple Bond

**DOI:** 10.1021/acs.inorgchem.1c00986

**Published:** 2021-05-17

**Authors:** Abraham B. Alemayehu, Laura J. McCormick-McPherson, Jeanet Conradie, Abhik Ghosh

**Affiliations:** † Department of Chemistry, UiT—The Arctic University of Norway, N-9037 Tromsø, Norway; ‡ Advanced Light Source, Lawrence Berkeley National Laboratory, Berkeley, California 94720-8229, United States; § Department of Chemistry, University of the Free State, P.O. Box 339, Bloemfontein 9300, Republic of South Africa

## Abstract

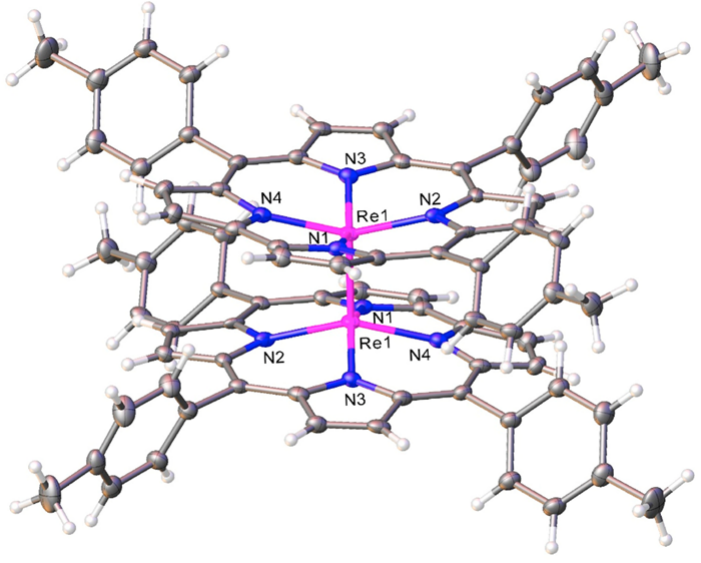

The interaction of free-base triarylcorroles with Re_2_(CO)_10_ in 1,2-dichlorobenzene in the presence of 2,6-lutidine at 180 °C under strict anerobic conditions afforded approximately 10% yields of rhenium corrole dimers. The compounds exhibited diamagnetic ^1^H NMR spectra consistent with a metal–metal quadruple bond with a σ^2^π^4^δ^2^ orbital occupancy. One of the compounds proved amenable to single-crystal X-ray structure determination, yielding a metal–metal distance of ∼2.24 Å, essentially identical to that in triple-bonded osmium corrole dimers. On the other hand, the electrochemical properties of Re and Os corrole dimers proved to be radically different. Thus, the reduction potentials of the Re corrole dimers are some 800 mV upshifted relative to those of their Os counterparts. Stated differently, the Re corrole dimers are dramatically easier to reduce, reflecting electron addition to δ* versus π* molecular orbitals for Re and Os corrole dimers, respectively. The data also imply electrochemical HOMO-LUMO gaps of only 1.0–1.1 V for rhenium corrole dimers, compared with values of 1.85–1.90 V for their Os counterparts. These HOMO–LUMO gaps rank among the first such values reported for quadruple-bonded transition-metal dimers for any type of supporting ligand, porphyrin-type or not.

## Abstract 2

The first metal−metal quadruple-bonded metallocorrole dimers have been synthesized in the form of three rhenium *meso*-triarylcorrole dimers. The compounds exhibit electrochemical HOMO−LUMO gaps of 1.0−1.1 V, which is some 750 mV smaller than those of their triple-bonded Os counterparts.

## Introduction

The interpretation of the very short Re–Re distance in the [Re_2_Cl_8_]^2–^ dianion^1,2^ as indicative of a metal–metal quadruple bond by Cotton in 1965 stands as a landmark in the history of chemical bonding.^3−7^ The novel feature of such a bond is a δ-orbital interaction, in addition to a σ and two π interactions. Subsequently, it became clear that the δ interaction makes only a small contribution to the metal–metal interaction energy and has next to no effect on the metal–metal distance.^8^ Nevertheless, the δ interaction has major implications for many physicochemical properties and especially for redox chemistry. Many of these insights originated from Collman and Arnold’s extensive studies of 4d and 5d metalloporphyrin dimers.^9^ For example, temperature-dependent ^1^H NMR studies of molybdenum and tungsten porphyrin dimers provided some of the first estimates of the strength of the δ interaction.^10−12^ Likewise, resonance Raman studies of molybdenum, rhenium, and osmium porphyrin dimers provided some of the first insights into the vibrational characteristics of metal–metal multiple bonds.^13^ Remarkably, in spite of sustained attention over decades, significant questions remain relative to the energetics of δ bonds. (a) How much are typical δ−δ* transition energies, especially as a function of different metals? (b) How much are typical singlet–triplet gaps? (c) What about the electrochemical HOMO-LUMO gaps? The last, in theory, would appear to be a simple matter; in practice, few quadruple-bonded systems exhibit clean, reversible reductions in their cyclic voltammograms, thwarting a simple approach to answering the question. Here we report a new class of quadruple-bonded systems in the form of three rhenium *meso*-triarylcorrole dimers, one of which was characterized via single-crystal X-ray diffraction analysis. Electrochemical studies and density functional theory (DFT) calculations on the complexes have now provided some of the clearest answers yet to the above questions,^14,15^ as outlined below.

## Results and Discussion

### Synthesis and Proof of Structure

**Scheme 1:**
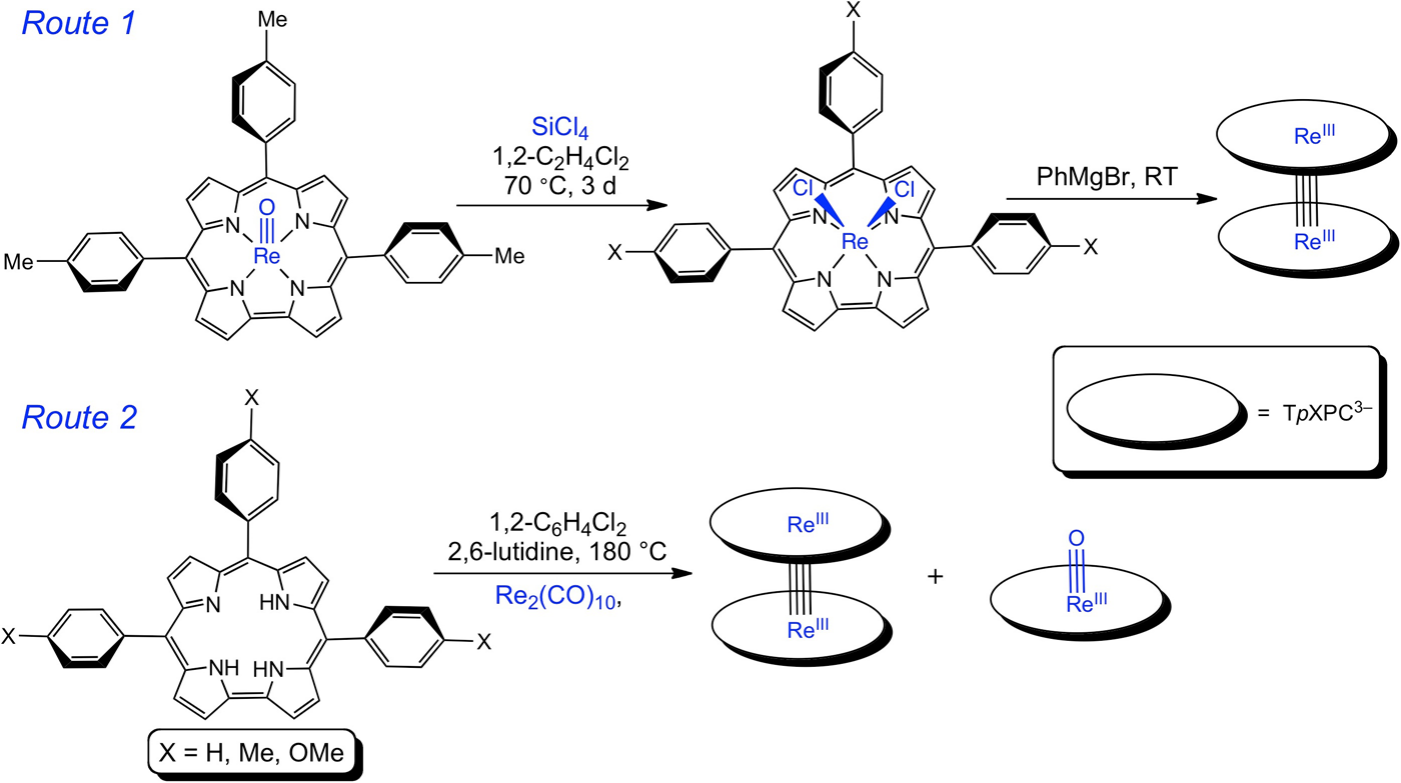
Two Routes to Rhenium Corrole Dimers

Ironically, given our long-standing interest in metal–metal multiple bonds,^16,17^ we stumbled upon the first quadruple-bonded metallocorrole dimer through sheer serendipity. Attempted derivatization of an ReCl_2_ Viking helmet corrole (generated as described by Bröring and co-workers^18^) with PhMgBr failed to yield the expected RePh_2_ product; mass spectrometric (MS) analysis of the products instead showed the presence of small quantities of what appeared to be a rhenium corrole dimer (Scheme 1). A further lucky break came, even before spectroscopic data were in place, in the form of a single-crystal X-ray diffraction structure, providing definitive proof of the formation of a multiple-bonded metallocorrole dimer.

Understandably, we sought a more rational route to the novel product. The interaction of free-base *meso*-tris(*p*-X-phenyl)corroles,^19,20^ H_3_[T*p*CH_3_PC] (X = H, Me, OMe), with Re_2_(CO)_10_ in 1,2-dichlorobenzene in the presence of 2,6-lutidine at 150 °C under strictly anerobic conditions yielded traces of the dimers {Re[T*p*XPC]}_2_ along with significant amounts of Re[T*p*XPC](O),^21^ the latter a testament to the exceedingly oxophilic nature of rhenium (Scheme 1). Increasing the temperature appeared to improve the yield of the dimer; ultimately, reflux conditions (i.e., a temperature of 180 °C) were considered optimum, which reliably led to >10% yields of the dimer. The Re corrole dimers could be readily separated from the ReO corroles via column chromatography so the synthesis, in spite of the low yields, proved simple and untedious in practice.

**Figure 1:**
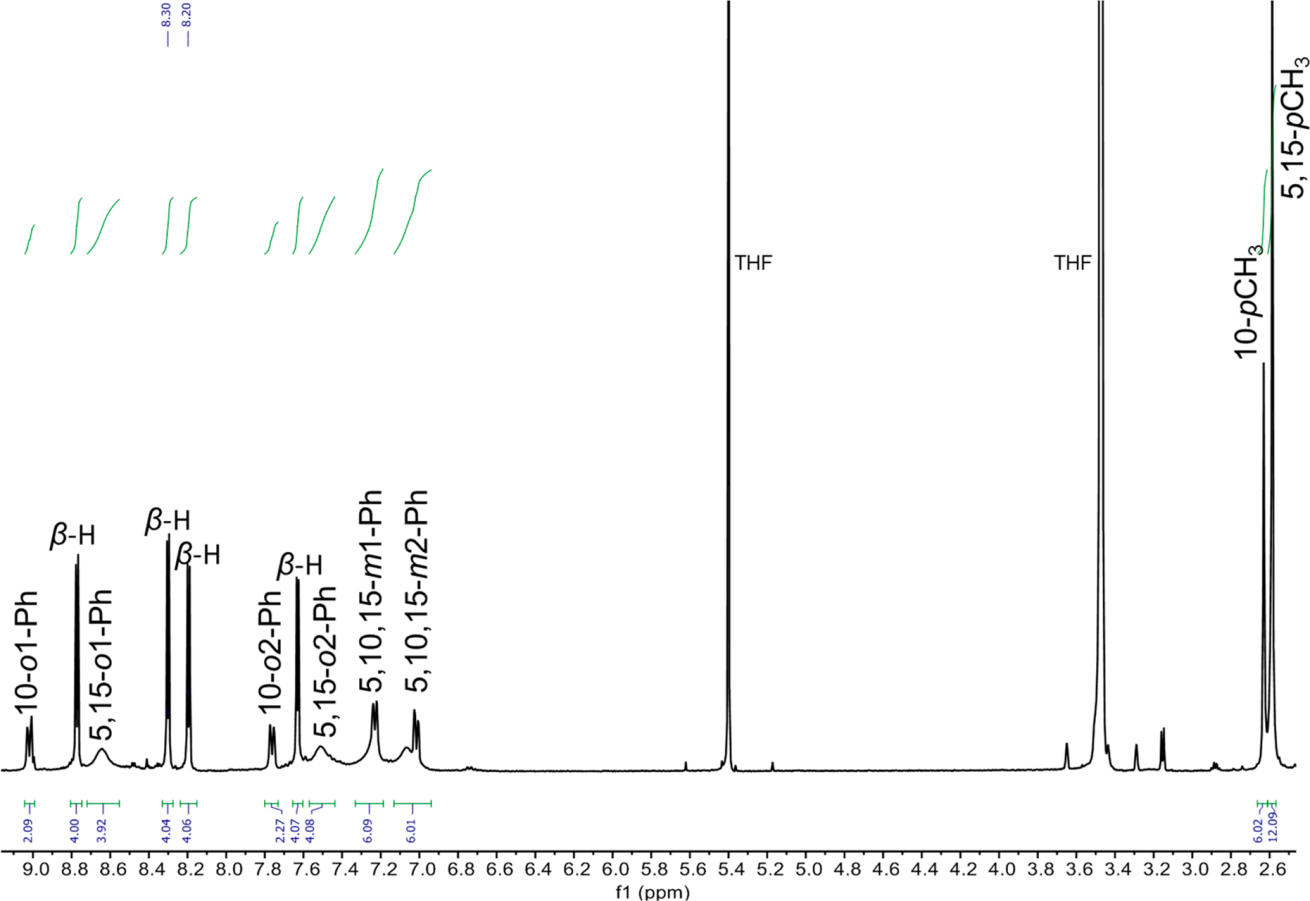
^1^H NMR spectrum of {Re[T*p*MePC]}_2_ in THF-*d*_8_ at 298 K.

The Re corrole dimers yielded reasonably sharp ^1^H NMR spectra at room temperature (Figure 1), providing unambiguous proof that the compounds are diamagnetic, consistent with a σ^2^π^4^δ^2^ quadruple-bonded description. The spectra could be essentially fully assigned, revealing symmetry-related *meso*-triarylcorrole ligands in which the ortho and meta protons of each phenyl ring are split into symmetry-distinct pairs, as is typical for square-pyramidally coordinated corrole derivatives.^21−24^ Unfortunately, the complex temperature-dependent dynamic behavior of the compounds prevented us from investigating the energetics of corrole rotation about the metal–metal axis, as was previously accomplished for Mo and W porphyrin dimers.^10−12^

**Figure 2:**
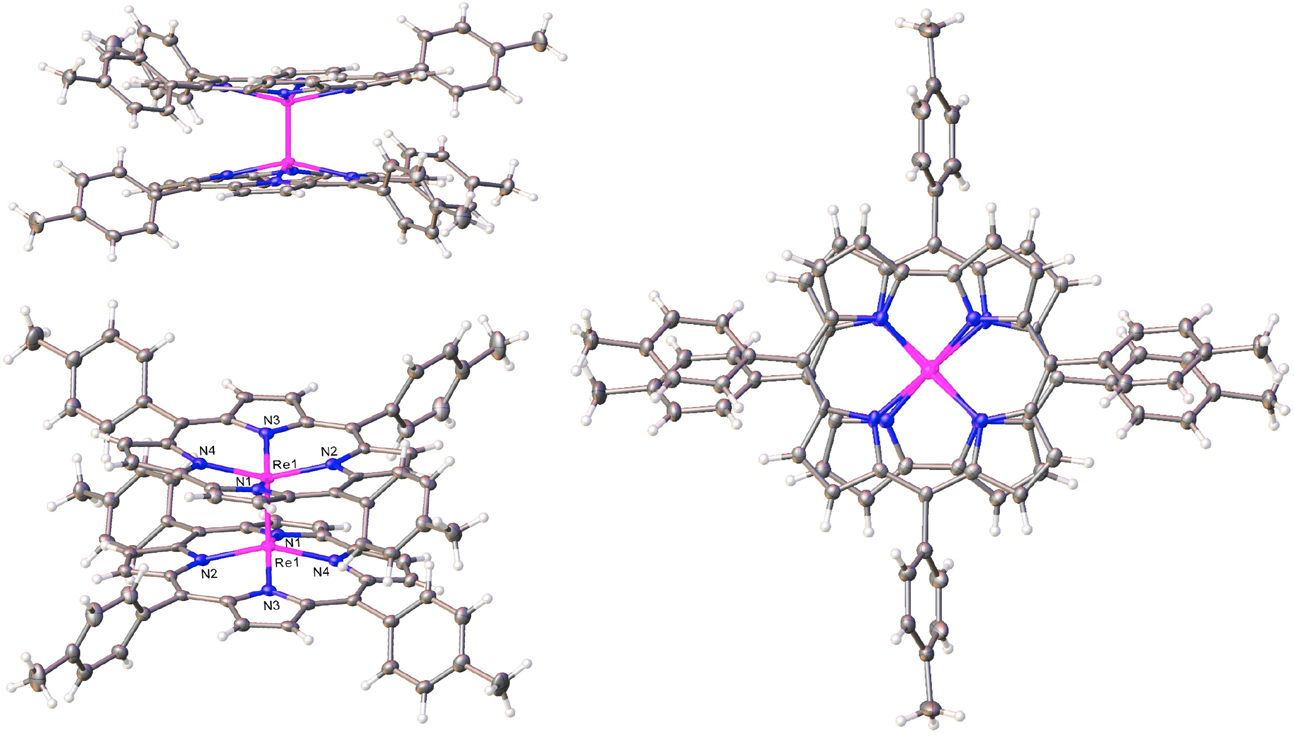
Thermal ellipsoid plots depicting three perspectives of {Re[T*p*MePC]}_2_. Selected distances (Å): Re1–N1 2.010(8), Re1–N2 1.999(9), Re1–N3 2.030(9), Re1–N4 1.997(9), Re1–Re1 2.2364(6).

The X-ray structure of {Re[T*p*MePC]}_2_ (space group *Pbcn*; Table 1 and Figure 2) indicated a dimer conformation with a crystallographically imposed center of symmetry: the preference for such a conformation is readily understandable in that it averts steric interactions between the 10-aryl groups. The metal–metal distance was found to be 2.236 Å, essentially identical with that found for Os corrole dimers.^17^ The distance is also very close to twice Pyykkö’s triple-bond covalent radius for Re (1.10 Å),^25^ proving (as elsewhere in the literature^7,9^) that the δ bond has little impact on the metal–metal distance. In other respects, the coordination geometry is unremarkable, with metal–nitrogen distances hovering around 2.00 Å, essentially the same as those in ReO corroles and a couple of hundredths of an angström longer than those in Os corrole dimers.^17^ Finally, the Re atom in {Re[T*p*MePC]}_2_ is displaced by about 0.531 Å above the mean N_4_ plane of the corrole, comparable to the analogous displacement of the metal in ReO^21^ and OsN^26^ corroles as well as in Os corrole dimers.^17^

**Table 1:** Crystallographic Data for {Re[T*p*CH_3_PC]}_2_

chemical formula	C_82_H_62_N_8_Cl_4_Re_2_
formula mass	1673.59
crystal system	orthorhombic
crystal size (mm^3^)	0.520 × 0.060 × 0.030
space group	*Pbcn*
λ (Å)	0.7749
*a* (Å)	14.9614(9)
*b* (Å)	20.5775(13)
*c* (Å)	21.8546(13)
α (deg)	90
β (deg)	90
γ (deg)	90
*Z*	4
*V* (Å^3^)	6728.3(7)
temperature (K)	100(2)
density (g/cm^3^)	1.652
no. of measured reflns.	368715
no. of unique reflns.	8721
no. of parameters	436
no. of restraints	0
*R*_int_	0.0772
*θ* range (deg)	2.032–31.617
*R*_1_, *wR*_2_ all data	0.1094, 0.2288
*S* (GOF) all data	1.077
max/min residual density (e/Å^3^)	3.026/–2.096

### UV–Vis Spectroscopy and Electrochemistry

**Figure 3:**
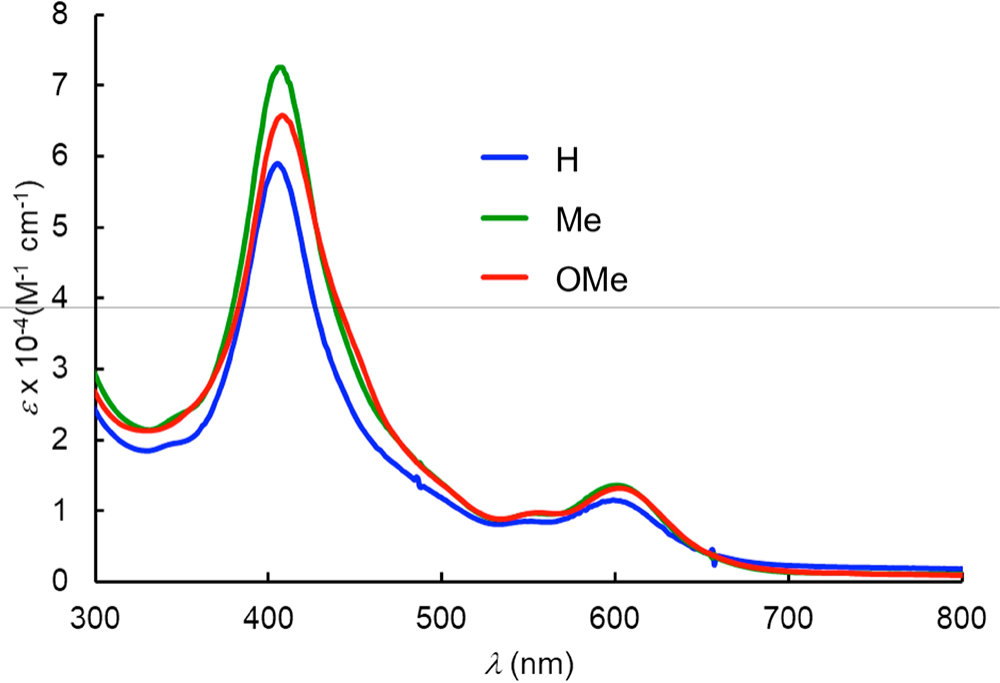
UV–vis spectra of {Re[T*p*XPC]}_2_ in dichloromethane for X = H (blue), Me (green), and OMe (red).

The optical spectra of the Re corrole dimers (Table 2 and Figure 3) proved rather unremarkable and qualitatively similar to those of their Os counterparts, with reasonably sharp Soret (405–408 nm) and Q (599–602 nm) bands. These are, however, hypsochromically and bathochromically shifted respectively relative to the main Soret (439–441 nm) and Q (585–592 nm) bands of the corresponding ReO corroles.^21^ No near-IR bands were evident up to 1200 nm.

**Table 2:** Spectroscopic and Electrochemical Properties of {Re[T*p*XPC]}_2_, {Ru[T*p*XPC]}_2_, and {Os[T*p*XPC]}_2_: Soret and Q Band λ_max_ (nm) and *E*_1/2_ Values (V vs SCE)

	λ_max_ (nm)									
complex	Soret	Q	*E*_1/2ox4_	*E*_1/2ox3_	*E*_1/2ox2_	*E*_1/2ox1_	*E*_1/2red1_	*E*_1/2red2_	Δ*E*	ref^a^
{Re[TPC]}_2_	405	599		1.18	0.84	0.57	–0.54	b	1.11	tw
{Re[T*p*MePC]}_2_	407	601		1.12	0.78	0.51	–0.55	b	1.06	tw
{Re[T*p*OMePC]}_2_	408	602		1.09	0.74	0.49	–0.57	b	1.06	tw
{Ru[T*p*CF_3_PC]}_2_	328, 397	541		1.31	1.09	0.76	–0.63	–1.43	1.39	(16)
{Ru[TPC]}_2_	328, 397	539	1.56	1.23	0.99	0.55	–0.86	–1.66	1.41	(16)
{Ru[T*p*MePC]}_2_	329, 398	538	1.44	1.18	0.98	0.52	–0.85		1.37	(16)
{Ru[T*p*OMePC]}_2_	329, 406	533	1.33	1.14	0.92	0.50	–0.86		1.36	(16)
{Os[T*p*CF_3_PC]}_2_	287, 407	583		1.28	1.01	0.79	–1.13	–1.54	1.92	(17)
{Os[TPC]}_2_	287, 405	584		1.15	0.93	0.60	–1.29	–1.69	1.89	(17)
{Os[T*p*MePC]}_2_	287, 407	584	1.35	1.09	0.88	0.55	–1.31	–1.72	1.86	(17)
{Os[T*p*OMePC]}_2_	286, 407	585	1.28	1.05	0.85	0.54	–1.32	–1.73	1.86	(17)

a tw = this work.

b A second reduction is partially discernible below −1.80 V, but it is not fully reversible at room temperature.

**Figure 4:**
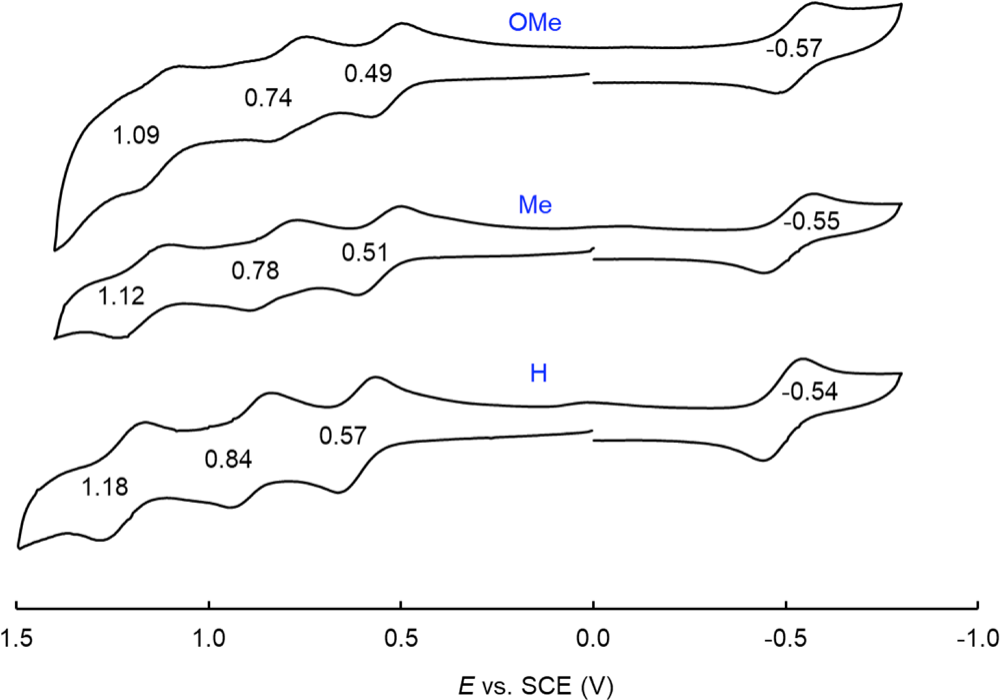
Cyclic voltammograms (V vs SCE) of {Re[T*p*XPC]}_2_ for X = OMe, Me, and H in dichloromethane containing 0.1 M TBAP. Scan rate = 100 mV/s.

**Figure 5:**
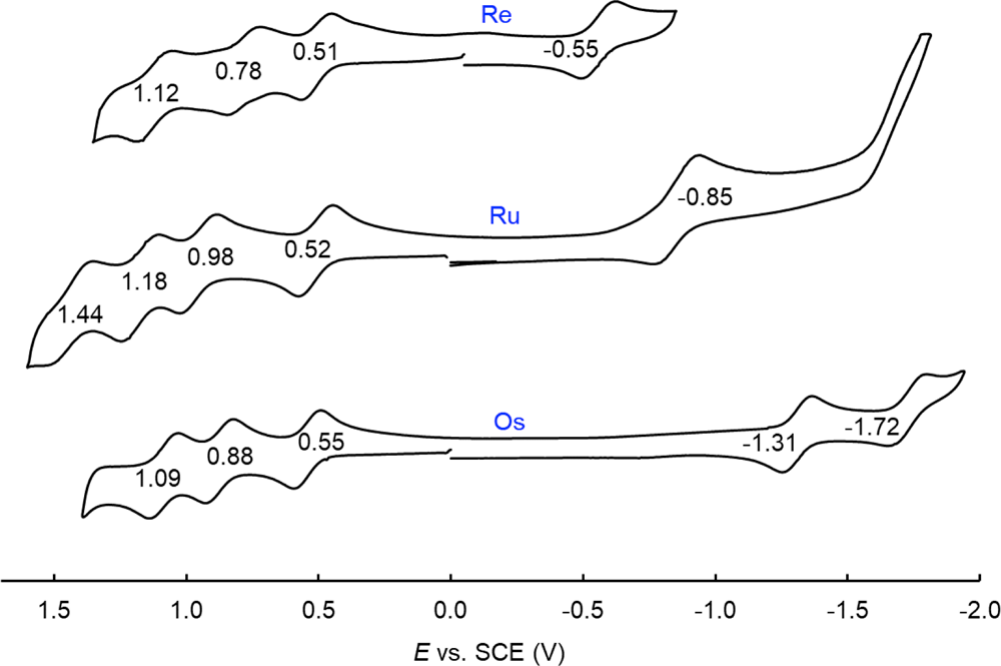
Comparison of the cyclic voltammograms of {M[T*p*MePC]}_2_ for M = Re, Ru, and Os.

Cyclic voltammetry, on the other hand, uncovered major differences among Re, Ru,^16,27,28^ and osmium^17^ corrole dimers (Table 2 and Figures 4 and 5).^29^ The three classes of complexes all exhibit at least three reversible oxidations and one reversible reduction. Furthermore, for a given corrole ligand, the oxidation potentials were found to be nearly identical for the three metals, suggesting a lack of sensitivity to the nature of the metal–metal bonding and, hence, ligand-centered oxidation. The reduction potentials, in contrast, proved to be dramatically different for the three metals, being algebraically in the order Re > Ru > Os. The nearly 750-mV difference between the reduction potentials of Re and Os corrole dimers appears to be consistent with the thermodynamic ease of electron addition into the δ* orbital of the former and the difficulty of electron addition to the much higher-energy π* orbital of the latter.^30^ For Ru, the reduction potential is not quite as negative as that for Os, in large part because an Ru–Ru π* LUMO is less relativistically destabilized than its Os counterpart. The electrochemical HOMO–LUMO gaps, i.e., the algebraic difference between the oxidation and reduction potentials, accordingly, are dramatically different for the three metals, increasing in the order Re < Ru < Os. Importantly, reversible reductions have rarely been observed for quadruple-bonded systems; the present study thus provides a unique measurement of the electrochemical HOMO–LUMO gap for such a system.

### DFT Calculations

Scalar-relativistic DFT (OLYP/ZORA-STO-TZ2P) calculations^14^ were undertaken on the ground state (*C*_2*h*_, *S* = 0), lowest triplet state (*C*_2_, *S* = 1), and cationic and anionic states (each *C*_2_, *S* = ^1^/_2_) of {Re[Cor]}_2_. The calculations revealed a small HOMO–LUMO gap (0.26 eV) that closely matched vertical S_0_–S_1_ (0.28) and S_0_–T_1_ (0.24 eV) gaps obtained from time-dependent DFT calculations. The adiabatic S_0_–T_1_ gap proved smaller, about 0.10 eV, in part reflecting the rotation^31^ of the two corrole rings relative to each other and the breaking of the δ bond. All of these energy gaps are considerably smaller than the electrochemical HOMO–LUMO gaps discussed above. That in itself, while interesting, is not particularly concerning, especially given the neglect of spin–orbit coupling in these calculations.

**Figure 6:**
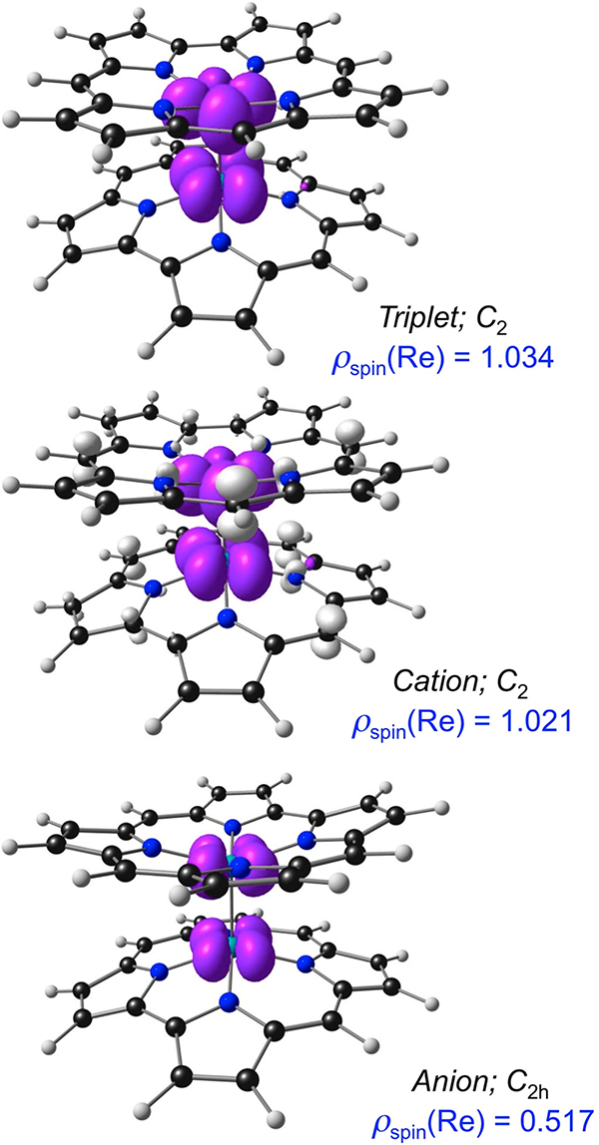
Spin-density profiles for the triplet, cationic, and anionic states of {Re[Cor]}_2_. The majority and minority spin densities are shown in violet and ivory, respectively. Also shown are the effective point groups.

The major contribution of the calculations relates to the nature of the ionized states of {Re[Cor]}_2_. While the calculated adiabatic ionization potential (5.88 eV) proved unremarkable (comparable to that of a variety of electron-rich porphyrin derivatives^32−36^), the electron affinity proved to be remarkably high (2.37 eV), indicating an unusually stable anionic state and in qualitative accord with the electrochemical data. Unsurprisingly, the spin density of the anionic state was found to correspond to electron addition to the δ* orbital of the neutral dimer (Figure 6).^31^ The nature of the cationic state proved to be more intriguing. For the {Re[Cor]}_2_ cation, *each* Re atom was found to carry just over one electron spin, while the two corroles together were found to carry just over one minority spin. In other words, the overall electronic configuration appears to be δ(↑)δ*(↑)π(↓), indicating a locally excited Re(III)–Re(III) axis antiferromagnetically coupled to a π radical spanning both corroles.^37−42^ Such a description presumably reflects the close spacing of molecular orbitals in the HOMO region of the neutral *C*_2*h*_ dimer and a pseudo-Jahn–Teller distortion leading to a *C*_2_ cation.^43^ Thus, the electrochemical HOMO–LUMO gap does not quite correspond to the δ−δ* orbital energy gap but may be legitimately regarded as an approximation to the latter.

## Conclusion

Porphyrin ligands permitted the synthesis of a variety of highly stable complexes with metal–metal multiple bonds, in turn permitting a variety of unique physicochemical measurements.^9−12^ Corroles now forcefully complement porphyrins in this role. The rhenium corrole dimers reported here are the first examples of quadruple-bonded metallocorrole dimers. The crystal structure of one such compound, {Re[T*p*MePC]}_2_, revealed an Re–Re distance of 2.24 Å, which is essentially identical to the Os–Os distance in triple-bonded osmium corrole dimers. Electrochemical studies, on the other hand, revealed dramatically higher (i.e., less negative) reduction potentials relative to the Os compounds, reflecting electron addition to a δ* LUMO in the Re case and to a π*-based LUMO in the Os case. These studies also indicate unusually small electrochemical HOMO–LUMO gaps of 1.0–1.1 V in the Re case; these, surprisingly, rank among the first such measurements reported for quadruple-bonded transition-metal dimers, with porphyrin-type supporting ligands or otherwise.

## Experimental Section

### Materials

Free-base corroles were synthesized via the so-called water–methanol method.^20^ All other reagents were purchased from Sigma-Aldrich (Merck) and used as received. Silica gel 60 (0.04–0.063 mm particle size, 230–400 mesh, Merck) was employed for flash chromatography. Silica gel 60 preparative thin-layer chromatographic plates (20 cm × 20 cm × 0.5 mm, Merck) were used for final purification of all complexes.

### Instrumental Methods

UV–vis spectra were recorded on a Cary 8454 spectrophotometer. ^1^H NMR spectra were recorded on a 400 MHz Bruker Avance III HD spectrometer (equipped with a 5-mm BB/^1^H SmartProbe) at a temperature of 298 K in tetrahydrofuran (THF)-*d*_8_ and referenced to residual THF protons at 3.48 and 2.37 ppm. High-resolution (HR) mass spectra were recorded (typically in the positive-ion mode) on a Thermo LTQ Orbitrap XL spectrometer equipped with an electrospray ION MAX source. Elemental analyses were performed by Atlantic Microlab, Inc.

Cyclic voltammetry was carried out at 298 K with an EG&G model 263A potentiostat having a three-electrode system: a glassy carbon working electrode, a platinum wire counterelectrode, and a standard calomel reference electrode (SCE). Tetra-*n*-butylammonium perchlorate, recrystallized twice from absolute ethanol and dried in a desiccator for at least two weeks, was used as the supporting electrolyte. Anhydrous CH_2_Cl_2_ (Sigma-Aldrich) was used as a solvent. The reference electrode was separated from the bulk solution by a fritted-glass bridge filled with a saturated AgCl/KCl mixture. The electrolyte solution was purged with argon for at least 2 min, and all measurements were carried out under an argon blanket. All potentials were referenced to the SCE.

### General Procedure for the Synthesis of {Re[T*p*XPC]}_2_

To a two-necked 50-mL round-bottom flask were added the free-base corrole H_3_[T*p*XPC], where X = H, CH_3_, and OCH_3_ (0.13 mmol), Re_2_(CO)_10_ (0.26 mmol, 173 mg), 2,6-lutidine (0.1 mL), 1,2-dichlorobenzene (10 mL), and a magnetic stirring bar. The contents of the mixture were deoxygenated with a constant flow of argon for 10 min and subsequently heated (refluxed) at 180 °C for 4 h, with constant stirring under argon. Completion of the reaction was indicated by disappearance of the Soret absorption of the free-base corrole. Upon cooling, the reaction mixture was loaded directly onto a silica-gel column with *n*-hexane as the mobile phase. 1,2-Dichlorobenzene was first removed by eluting with pure *n*-hexane. Subsequently, 1:3 *n*-hexane/dichloromethane mixtures were used to elute the red ReO corrole and then the green Re corrole dimer. The Re corrole dimers were further purified via preparative thin-layer chromatography using 1:2 *n*-hexane/dichloromethane, giving final yields of >10%. Analytical details for the new compounds are as follows.

### {Re[TPC]}_2_

Yield: 11 mg (11.65%). UV–vis [CH_2_Cl_2_; λ_max_, nm (ε, ×10^–4^ M^–1^ cm^–1^)]: 405 (5.90), 548 (0.85), 599 (1.15). ^1^H NMR (400 MHz, 25 °C, THF-*d*_8_): δ 9.02 (d, 4H, ^3^*J*_HH_ = 4.00 Hz, β-H), 8.86 (bs, 4H, β-H), 8.54 (d, 4H, ^3^*J*_HH_ = 4.04 Hz, β-H), 8.37 (bs, 4H, β-H), 8.17 (bs, 4H, 5,15-*o*1-Ph), 8.07 (bs, 2H, 10-*o*1-Ph), 7.81 (m, 6H, 5,10,15-*o*2-Ph), 7.75 (bs, 6H, 5,10,15-*p*-Ph), 7.60 (m, 6H, 5,10,15-*m*1-Ph), 7.32 (m, 6H, 5,10,15-*m*2-Ph). Elem anal. found: C, 62.99; H, 3.62; N, 7.54. Calcd: C, 62.61; H, 3.27; N, 7.89. MS (ESI): M^+^ = 1420.2979 (expt), 1420.2965 (calcd for C_74_H_46_N_8_Re_2_).

### {Re[T*p*MePC]}_2_

Yield: 10.2 mg (10.43%). UV–vis [CH_2_Cl_2_; λ_max_, nm (ε, ×10^–4^ M^–1^ cm^–1^)]: 407 (7.25), 553 (0.96), 601 (1.36). ^1^H NMR (400 MHz, 25 °C, THF-*d*_8_): δ 9.02 (d, 2H, ^3^*J*_HH_ = 9.00 Hz, 10-*o*1-Ph), 8.77 (d, 4H, ^3^*J*_HH_ = 4.60 Hz, β-H), 8.64 (bs, 4H, 5,15-*o*1-Ph), 8.30 (d, 4H, ^3^*J*_HH_ = 4.16 Hz, β-H), 8.19 (d, 4H, ^3^*J*_HH_ = 4.56 Hz, β-H), 7.76 (d, 2H, ^3^*J*_HH_ = 7.52 Hz, 10-*o*2-Ph), 7.63 (d, 4H, ^3^*J*_HH_ = 4.16 Hz, β-H), 7.51 (bs, 4H, 5,15-*o*2-Ph), 7.23 (d, 6H, ^3^*J*_HH_ = 7.04 Hz, 5,10,15-*m*1-Ph), 7.13 (m, 6H, 5,10,15-*m*2-Ph), 2.63 (s, 6H, 10-*p*CH_3_), 2.59 (s, 12H, 5,15-*p*CH_3_). Elem anal. found: C, 63.99; H, 3.68; N, 7.34. Calcd: C, 63.90; H, 3.89; N, 7.05. MS (ESI): M^+^ = 1504.3918 (expt), 1504.3906 (calcd for C_80_H_58_N_8_Re_2_).

### {Re[T*p*OMePC]}_2_

Yield: 12 mg (11.53%). UV–vis [CH_2_Cl_2_; λ_max_, nm (ε, ×10^–4^ M^–1^ cm^–1^)]: 408 (6.57), 554 (0.92), 602 (1.31). ^1^H NMR (400 MHz, 25 °C, THF-*d*_8_): δ 9.04 (d, 2H, ^3^*J*_HH_ = 8.72 Hz, 10-*o*1-Ph), 8.80 (d, 4H, ^3^*J*_HH_ = 4.56 Hz, β-H), 8.63 (bs, 4H, 5,15-*o*1-Ph), 8.29 (d, 4H, ^3^*J*_HH_ = 4.16 Hz, β-H), 8.19 (d, 4H, ^3^*J*_HH_ = 3.40 Hz, β-H), 7.60 (bs, 4H, 5,15-*o*2-Ph), 7.54 (d, 4H, ^3^*J*_HH_ = 8.56 Hz, β-H), 7.28 (bs, 6H, Ph), 7.16–6.96 (m, 8H, Ph), 4.03 (s, 6H, 10-*p*OCH_3_), 3.99 (s, 12H, 5,15-*p*OCH_3_). Elem anal. found: C, 60.35; H, 3.72; N, 7.34. Calcd: C, 59.98; H, 3.61; N, 7.00. MS (ESI): M^+^ = 1600.3617 (expt), 1600.3601 (calcd for C_80_H_58_O_6_N_8_Re_2_).

### Crystallization and Crystallography

X-ray data were collected on beamline 11.3.1 at the Advanced Light Source of Lawrence Berkeley National Laboratory, Berkeley, CA. The samples were mounted on MiTeGen kapton loops and placed in a 100(2) K nitrogen cold stream provided by an Oxford Cryostream 700 Plus low-temperature apparatus on the goniometer head of a Bruker D8 diffractometer equipped with a PHOTONII CPAD detector. Diffraction data were collected using synchrotron radiation monochromated with silicon (111) to a wavelength of 0.7749(1) Å. An approximate full-sphere of data was collected using 1° ω scans. The structures were solved by intrinsic phasing methods (*SHELXT*)^44^ and refined by full-matrix least squares on *F*^2^ (*SHELXL-2018*).^45^ H atoms were geometrically calculated and refined as riding atoms.

### Computational Methods

DFT calculations were carried out with the *ADF 2018* program system.^46^ Relativistic effects were taken into account with the zeroth-order regular approximation (ZORA^47^) to the Dirac equation applied as a scalar correction. Specially optimized all-electron ZORA STO-TZ2P basis sets were used throughout. A variety of exchange-correlation functionals were tested; the results quoted are those for OLYP,^48,49^ one of the better generalized gradient approximations that we have extensively used in our studies of metalloporphyrin-type compounds.^14^

## Supporting Information Available

^1^H NMR and HR-ESI-MS spectra and optimized Cartesian coordinates (PDF)

## Accession Codes

CCDC 2073973 contains the supplementary crystallographic data for this paper. These data can be obtained free of charge via www.ccdc.cam.ac.uk/data_request/cif, or by emailing data_request@ccdc.cam.ac.uk, or by contacting The Cambridge Crystallographic Data Centre, 12 Union Road, Cambridge CB2 1EZ, UK; fax: +44 1223 336033.
