# Supplementary material for: Rhenium Corrole Dimers: Electrochemical Insights into the Nature of the Metal–Metal Quadruple Bond
Source: Inorg Chem. 2021 May 17;60(11):8315–21. doi: 10.1021/acs.inorgchem.1c00986 (PMC8278387; doi:10.1021/acs.inorgchem.1c00986)
Supplement: Supplementary file 1 — ic1c00986_si_001.pdf [file ic1c00986_si_001.pdf]

Supporting Information

Rhenium Corrole Dimers: Electrochemical Insights into the Nature of the Metal-Metal Quadruple Bond

Abraham B. Alemayehu,[a] Laura J. McCormick-McPherson,[b] Jeanet Conradie,[a,c] and Abhik Ghosh*[,a]

[a] Department of Chemistry, UiT – The Arctic University of Norway, N-9037 Tromsø, Norway;

Email: abhik.ghosh@uit.no; Telephone: +47 45476145

[b]Advanced Light Source, Lawrence Berkeley National Laboratory, Berkeley, CA 94720-8229, USA

[c]Department of Chemistry, University of the Free State, P.O. Box 339, Bloemfontein 9300, Republic of South Africa.

**Contents**	**Page**
A. Additional spectroscopic data	S2
B. Optimized Cartesian coordinates	S5

A. Additional spectroscopic data

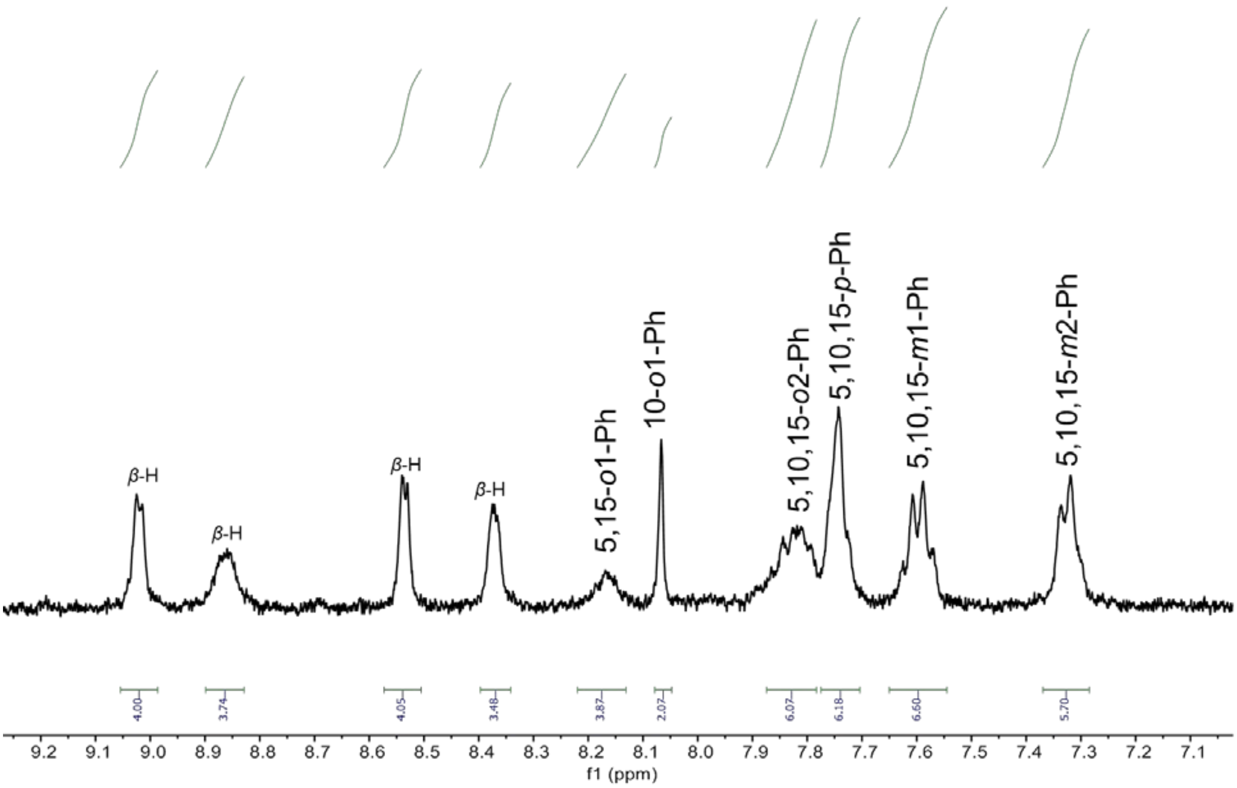

Figure S1. ^{1}H NMR spectrum of {Re[TPC]}$_2$ in THF at 298 K.

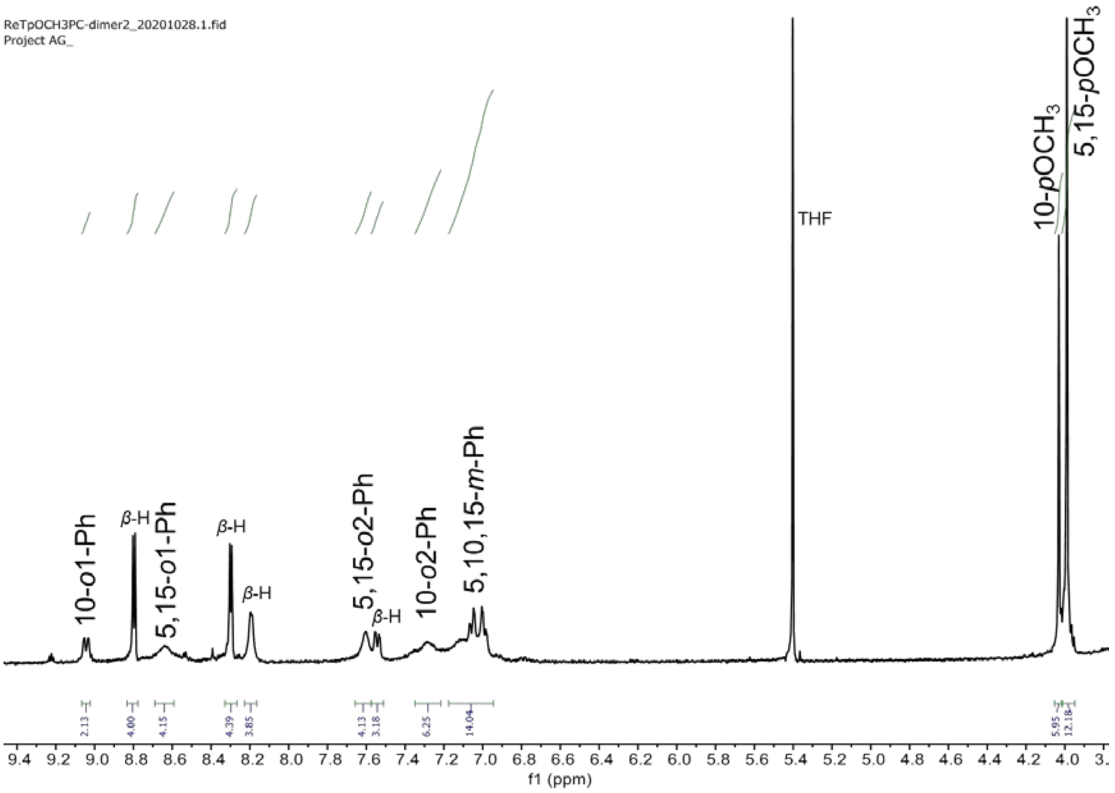

Figure S2. ^{1}H NMR spectrum of {Re[T*p*OMePC]}$_2$ in THF at 298 K.

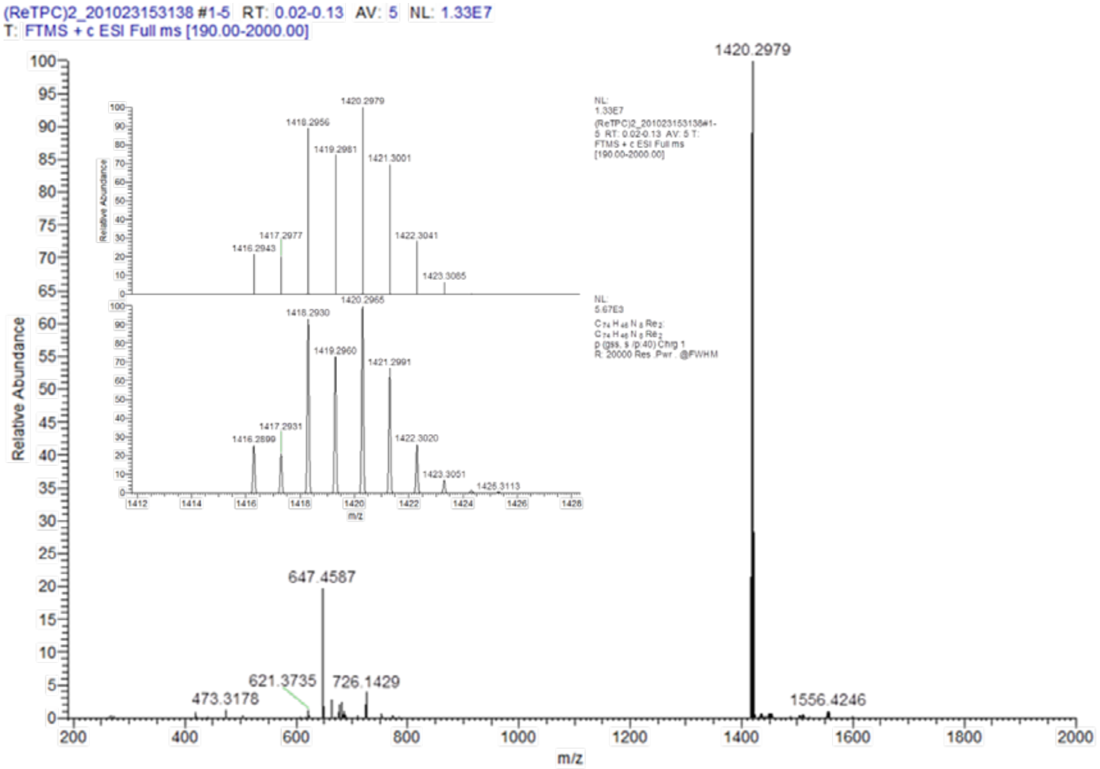

Figure S3. Postive-mode ESI-MS of {Re[TPC]}2. Inset: isotope pattern for molecular ion – experimental (top) and simulation (bottom).

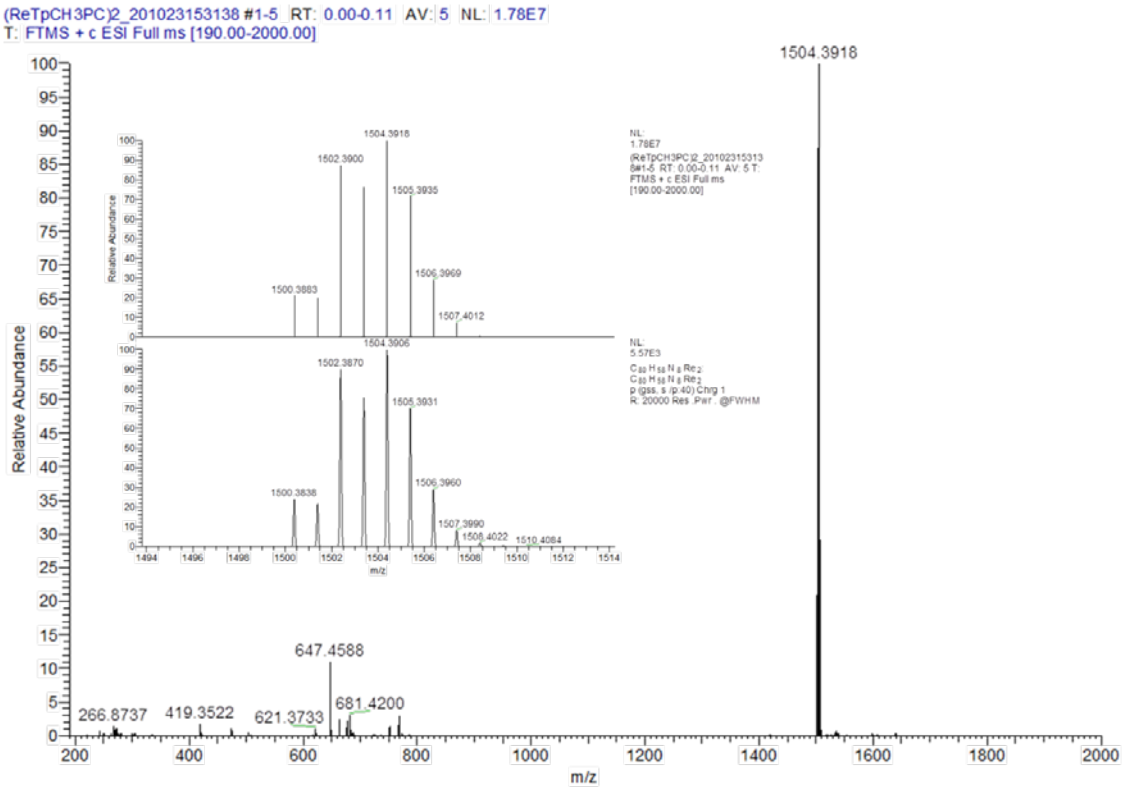

Figure S4. Postive-mode ESI-MS of {Re[T*p*MePC]}2. Inset: isotope pattern for molecular ion – experimental (top) and simulation (bottom).

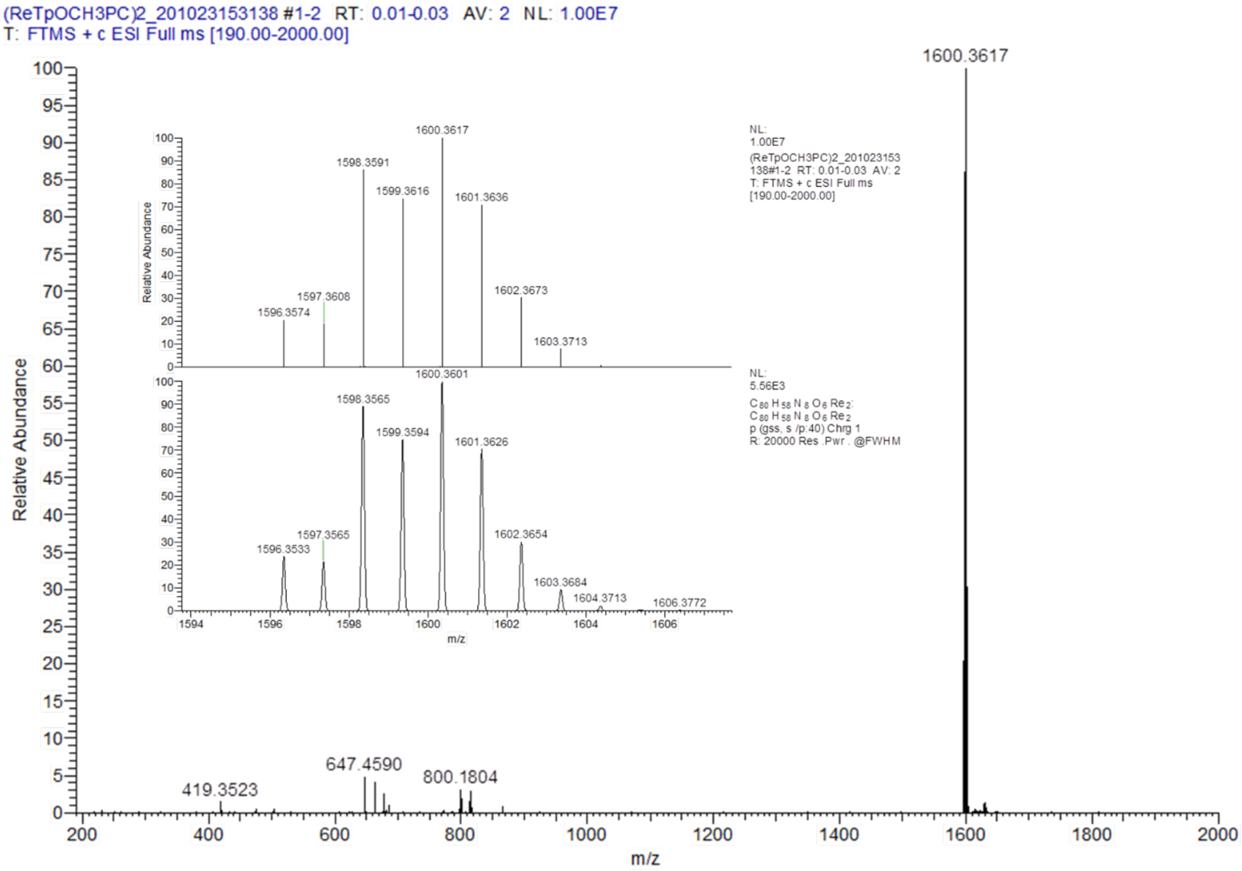

Figure S5. Postive-mode ESI-MS of {Re[T*p*OMePC]}$_2$. Inset: isotope pattern for molecular ion – experimental (top) and simulation (bottom).

B. Optimized OLYP/ZORA-STO-TZ2P Cartesian coordinates (Å)

{Re[Cor]}$_2$; *S* = 0; *C*$_{2h}$ symmetry constraint

C	0.154028000	2.038259000	2.819067000
C	0.154028000	2.038259000	-2.819067000
C	0.762458000	-2.908675000	3.492654000
C	0.762458000	-2.908675000	-3.492654000
C	1.199152000	-2.999179000	1.260095000
C	1.199152000	-2.999179000	-1.260095000
C	1.262711000	1.362343000	3.346543000
C	1.262711000	1.362343000	-3.346543000
C	1.581296000	-3.490269000	2.550995000
C	1.581296000	-3.490269000	-2.550995000
C	1.685140000	-3.377085000	0.000000000
C	2.253206000	0.699184000	2.602999000
C	2.253206000	0.699184000	-2.602999000
C	3.344930000	0.077801000	0.711884000
C	3.344930000	0.077801000	-0.711884000
C	3.550087000	0.215549000	2.966923000
C	3.550087000	0.215549000	-2.966923000
C	4.221306000	-0.152388000	1.803630000
C	4.221306000	-0.152388000	-1.803630000
C	-0.154028000	-2.038259000	2.819067000
C	-0.154028000	-2.038259000	-2.819067000
C	-0.762458000	2.908675000	3.492654000
C	-0.762458000	2.908675000	-3.492654000
C	-1.199152000	2.999179000	1.260095000
C	-1.199152000	2.999179000	-1.260095000
C	-1.262711000	-1.362343000	3.346543000
C	-1.262711000	-1.362343000	-3.346543000
C	-1.581296000	3.490269000	2.550995000
C	-1.581296000	3.490269000	-2.550995000
C	-1.685140000	3.377085000	0.000000000
C	-2.253206000	-0.699184000	2.602999000
C	-2.253206000	-0.699184000	-2.602999000
C	-3.344930000	-0.077801000	0.711884000
C	-3.344930000	-0.077801000	-0.711884000
C	-3.550087000	-0.215549000	2.966923000
C	-3.550087000	-0.215549000	-2.966923000
C	-4.221306000	0.152388000	1.803630000
C	-4.221306000	0.152388000	-1.803630000
H	0.761301000	-3.089816000	4.560296000
H	0.761301000	-3.089816000	-4.560296000
H	1.415609000	1.443863000	4.418854000
H	1.415609000	1.443863000	-4.418854000
H	2.364583000	-4.218086000	2.720775000
H	2.364583000	-4.218086000	-2.720775000
H	2.496785000	-4.098852000	0.000000000
H	3.940944000	0.186784000	3.976036000

H	3.940944000	0.186784000	-3.976036000
H	5.236873000	-0.520179000	1.732769000
H	5.236873000	-0.520179000	-1.732769000
H	-0.761301000	3.089816000	4.560296000
H	-0.761301000	3.089816000	-4.560296000
H	-1.415609000	-1.443863000	4.418854000
H	-1.415609000	-1.443863000	-4.418854000
H	-2.364583000	4.218086000	2.720775000
H	-2.364583000	4.218086000	-2.720775000
H	-2.496785000	4.098852000	0.000000000
H	-3.940944000	-0.186784000	3.976036000
H	-3.940944000	-0.186784000	-3.976036000
H	-5.236873000	0.520179000	1.732769000
H	-5.236873000	0.520179000	-1.732769000
N	0.165249000	-2.085437000	1.462155000
N	0.165249000	-2.085437000	-1.462155000
N	2.154012000	0.553479000	1.235284000
N	2.154012000	0.553479000	-1.235284000
N	-0.165249000	2.085437000	1.462155000
N	-0.165249000	2.085437000	-1.462155000
N	-2.154012000	-0.553479000	1.235284000
N	-2.154012000	-0.553479000	-1.235284000
Re	0.590273000	0.927732000	0.000000000
Re	-0.590273000	-0.927732000	0.000000000

{Re[Cor]}$_2$; *S* = 1; *C*$_2$ symmetry constraint

C	0.153073000	1.824906000	2.982548000
C	0.300636000	1.832102000	-2.983901000
C	0.830148000	-2.392807000	3.854383000
C	1.039792000	-2.243082000	-2.954300000
C	1.205449000	1.820514000	-4.095035000
C	1.497457000	1.469322000	3.188159000
C	1.669163000	-2.389007000	1.745885000
C	1.822986000	-2.422787000	-1.800335000
C	1.939551000	-2.748427000	3.091676000
C	2.208992000	-2.552402000	0.438363000
C	2.375918000	1.289697000	-2.216063000
C	2.434048000	1.177084000	2.186586000
C	2.460072000	1.493424000	-3.631797000
C	3.079145000	-3.075461000	-1.591500000
C	3.307304000	-3.161245000	-0.219773000
C	3.330419000	0.990342000	0.098132000
C	3.409772000	1.033822000	-1.302136000
C	3.847252000	0.984696000	2.315346000
C	4.389907000	0.873573000	1.054242000
C	-0.153073000	-1.824906000	2.982548000
C	-0.300636000	-1.832102000	-2.983901000
C	-0.830148000	2.392807000	3.854383000
C	-1.039792000	2.243082000	-2.954300000

C	-1.205449000	-1.820514000	-4.095035000
C	-1.497457000	-1.469322000	3.188159000
C	-1.669163000	2.389007000	1.745885000
C	-1.822986000	2.422787000	-1.800335000
C	-1.939551000	2.748427000	3.091676000
C	-2.208992000	2.552402000	0.438363000
C	-2.375918000	-1.289697000	-2.216063000
C	-2.434048000	-1.177084000	2.186586000
C	-2.460072000	-1.493424000	-3.631797000
C	-3.079145000	3.075461000	-1.591500000
C	-3.307304000	3.161245000	-0.219773000
C	-3.330419000	-0.990342000	0.098132000
C	-3.409772000	-1.033822000	-1.302136000
C	-3.847252000	-0.984696000	2.315346000
C	-4.389907000	-0.873573000	1.054242000
H	0.697450000	-2.556215000	4.916448000
H	0.928332000	2.070038000	-5.111705000
H	1.479045000	-2.531313000	-3.904810000
H	1.872446000	1.500655000	4.206890000
H	2.836018000	-3.239909000	3.446926000
H	3.375003000	1.425143000	-4.206938000
H	3.710597000	-3.471902000	-2.376699000
H	4.150379000	-3.636380000	0.264995000
H	4.377803000	0.967673000	3.259167000
H	4.402003000	0.911723000	-1.726693000
H	5.434132000	0.745519000	0.797998000
H	-0.697450000	2.556215000	4.916448000
H	-0.928332000	-2.070038000	-5.111705000
H	-1.479045000	2.531313000	-3.904810000
H	-1.872446000	-1.500655000	4.206890000
H	-2.836018000	3.239909000	3.446926000
H	-3.375003000	-1.425143000	-4.206938000
H	-3.710597000	3.471902000	-2.376699000
H	-4.150379000	3.636380000	0.264995000
H	-4.377803000	-0.967673000	3.259167000
H	-4.402003000	-0.911723000	-1.726693000
H	-5.434132000	-0.745519000	0.797998000
N	0.413626000	-1.798254000	1.725495000
N	1.040103000	1.467151000	-1.857679000
N	1.351525000	-2.081729000	-0.548862000
N	2.145129000	1.137595000	0.819662000
N	-0.413626000	1.798254000	1.725495000
N	-1.040103000	-1.467151000	-1.857679000
N	-1.351525000	2.081729000	-0.548862000
N	-2.145129000	-1.137595000	0.819662000
Re	0.318090000	1.076279000	-0.016374000
Re	-0.318090000	-1.076279000	-0.016374000

{Re[Cor]}$_2$ cation; S = ½; C_2 symmetry constraint

C	0.067058000	1.883586000	2.975748000
C	0.379821000	1.763498000	-2.986328000
C	0.937983000	-2.479995000	3.808961000
C	0.967064000	-2.183502000	-2.997859000
C	1.305341000	1.719212000	-4.075289000
C	1.401444000	1.538714000	3.221854000
C	1.713449000	-2.427386000	1.679784000
C	1.779250000	-2.387215000	-1.874056000
C	2.021310000	-2.824959000	3.007871000
C	2.221207000	-2.567016000	0.345291000
C	2.373942000	1.225164000	2.246133000
C	2.425587000	1.224984000	-2.157716000
C	2.548555000	1.391411000	-3.572828000
C	3.036516000	-3.054292000	-1.713292000
C	3.298331000	-3.172832000	-0.350682000
C	3.324251000	0.986242000	0.194580000
C	3.435290000	0.989607000	-1.206406000
C	3.781687000	1.050775000	2.422966000
C	4.359835000	0.906623000	1.177736000
C	-0.067058000	-1.883586000	2.975748000
C	-0.379821000	-1.763498000	-2.986328000
C	-0.937983000	2.479995000	3.808961000
C	-0.967064000	2.183502000	-2.997859000
C	-1.305341000	-1.719212000	-4.075289000
C	-1.401444000	-1.538714000	3.221854000
C	-1.713449000	2.427386000	1.679784000
C	-1.779250000	2.387215000	-1.874056000
C	-2.021310000	2.824959000	3.007871000
C	-2.221207000	2.567016000	0.345291000
C	-2.373942000	-1.225164000	2.246133000
C	-2.425587000	-1.224984000	-2.157716000
C	-2.548555000	-1.391411000	-3.572828000
C	-3.036516000	3.054292000	-1.713292000
C	-3.298331000	3.172832000	-0.350682000
C	-3.324251000	-0.986242000	0.194580000
C	-3.435290000	-0.989607000	-1.206406000
C	-3.781687000	-1.050775000	2.422966000
C	-4.359835000	-0.906623000	1.177736000
H	0.831611000	-2.672957000	4.868878000
H	1.057105000	1.951419000	-5.103001000
H	1.376789000	-2.459412000	-3.965143000
H	1.754327000	1.606035000	4.246912000
H	2.921521000	-3.334793000	3.324621000
H	3.476858000	1.309489000	-4.123587000
H	3.640959000	-3.442978000	-2.522692000
H	4.146522000	-3.669153000	0.102208000
H	4.288263000	1.071729000	3.379388000
H	4.438675000	0.860838000	-1.602294000
H	5.412349000	0.786904000	0.954803000

H	-0.831611000	2.672957000	4.868878000
H	-1.057105000	-1.951419000	-5.103001000
H	-1.376789000	2.459412000	-3.965143000
H	-1.754327000	-1.606035000	4.246912000
H	-2.921521000	3.334793000	3.324621000
H	-3.476858000	-1.309489000	-4.123587000
H	-3.640959000	3.442978000	-2.522692000
H	-4.146522000	3.669153000	0.102208000
H	-4.288263000	-1.071729000	3.379388000
H	-4.438675000	-0.860838000	-1.602294000
H	-5.412349000	-0.786904000	0.954803000
N	0.467919000	-1.829477000	1.702424000
N	1.083638000	1.423321000	-1.834987000
N	1.342165000	-2.069843000	-0.600550000
N	2.118989000	1.149448000	0.879581000
N	-0.467919000	1.829477000	1.702424000
N	-1.083638000	-1.423321000	-1.834987000
N	-1.342165000	2.069843000	-0.600550000
N	-2.118989000	-1.149448000	0.879581000
Re	0.313491000	1.075686000	-0.004727000
Re	-0.313491000	-1.075686000	-0.004727000

{Re[Cor]}$_2$ anion; *S* = ½; *C*$_2$ symmetry constraint

C	0.150483000	1.926117000	-3.333669000
C	0.194194000	1.898644000	3.328984000
C	0.980596000	-2.188206000	2.608097000
C	1.015556000	-2.207908000	-2.595793000
C	1.389796000	1.527666000	-2.808469000
C	1.426468000	1.503814000	2.784882000
C	2.181323000	-2.875264000	2.980173000
C	2.190662000	-2.566727000	0.729756000
C	2.200775000	-2.570874000	-0.698804000
C	2.219417000	-2.901411000	-2.946130000
C	2.641035000	1.408017000	-3.503767000
C	2.686436000	1.375584000	3.462853000
C	2.916091000	-3.117134000	1.819729000
C	2.939356000	-3.132671000	-1.774185000
C	3.004229000	1.072392000	-1.284033000
C	3.020405000	1.059927000	1.235426000
C	3.619187000	0.931064000	-0.028798000
C	3.622442000	1.128089000	-2.580026000
C	3.655345000	1.103182000	2.523776000
C	-0.150483000	-1.926117000	-3.333669000
C	-0.194194000	-1.898644000	3.328984000
C	-0.980596000	2.188206000	2.608097000
C	-1.015556000	2.207908000	-2.595793000
C	-1.389796000	-1.527666000	-2.808469000
C	-1.426468000	-1.503814000	2.784882000
C	-2.181323000	2.875264000	2.980173000
C	-2.190662000	2.566727000	0.729756000

C	-2.200775000	2.570874000	-0.698804000
C	-2.219417000	2.901411000	-2.946130000
C	-2.641035000	-1.408017000	-3.503767000
C	-2.686436000	-1.375584000	3.462853000
C	-2.916091000	3.117134000	1.819729000
C	-2.939356000	3.132671000	-1.774185000
C	-3.004229000	-1.072392000	-1.284033000
C	-3.020405000	-1.059927000	1.235426000
C	-3.619187000	-0.931064000	-0.028798000
C	-3.622442000	-1.128089000	-2.580026000
C	-3.655345000	-1.103182000	2.523776000
H	0.121080000	2.119621000	-4.403131000
H	0.177690000	2.081192000	4.400629000
H	2.438896000	-3.186762000	3.985497000
H	2.488208000	-3.224183000	-3.944852000
H	2.765048000	1.545767000	-4.571602000
H	2.824451000	1.502378000	4.530342000
H	3.856520000	-3.650756000	1.751075000
H	3.877103000	-3.668497000	-1.688088000
H	4.681318000	0.992492000	-2.765352000
H	4.694327000	0.771582000	-0.036384000
H	4.716341000	0.964414000	2.694041000
H	-0.121080000	-2.119621000	-4.403131000
H	-0.177690000	-2.081192000	4.400629000
H	-2.438896000	3.186762000	3.985497000
H	-2.488208000	3.224183000	-3.944852000
H	-2.765048000	-1.545767000	-4.571602000
H	-2.824451000	-1.502378000	4.530342000
H	-3.856520000	3.650756000	1.751075000
H	-3.877103000	3.668497000	-1.688088000
H	-4.681318000	-0.992492000	-2.765352000
H	-4.694327000	-0.771582000	-0.036384000
H	-4.716341000	-0.964414000	2.694041000
N	1.054284000	-1.972880000	1.252446000
N	1.072442000	-1.979291000	-1.241609000
N	1.646732000	1.278841000	-1.462558000
N	1.665688000	1.267053000	1.433497000
N	-1.054284000	1.972880000	1.252446000
N	-1.072442000	1.979291000	-1.241609000
N	-1.646732000	-1.278841000	-1.462558000
N	-1.665688000	-1.267053000	1.433497000
Re	0.250376000	1.078600000	-0.005652000
Re	-0.250376000	-1.078600000	-0.005652000
